# Association Between Patient Age and Severity in Acute Appendicitis

**DOI:** 10.7759/cureus.83431

**Published:** 2025-05-03

**Authors:** Maria Vigil Escalera Bejarano, Elias Gallardo-Navarro, José Manuel Gomez López, Aldo Alejandro Tirado Cortes

**Affiliations:** 1 Department of General Surgery, Hospital Español, Mexico City, MEX

**Keywords:** abdominal pain, diagnosis of acute appendicitis, minimally invasive laparoscopy, robbins stages, senior patients

## Abstract

Objective

The objective of this study is to analyze and describe the relationship between patient age and the severity of acute appendicitis across its different stages, with the aim of stratifying the risk of post-surgical complications.

Methods

This retrospective, descriptive, observational study was conducted at a single center. A total of 106 patients underwent laparoscopic appendectomy over a period of two years. The variables analyzed included sex, age, Robbins stages, the presence of comorbidities, length of hospital stay, drainage use, and the presence of complications during hospitalization.

Results

A total of 106 patients underwent surgery, of which 49 (46.2%) were women and 57 (53.7%) were men. The mean age was 40 years, with a range from 13 to 80 years. The mean length of hospital stay was one day, ranging from zero to nine days. According to Robbins stages, 17.9% of patients (19) had stage IV acute appendicitis, 19.8% (21) had stage III, 57.6% (61) had stage II, and 4.7% (five) had stage I. Among the 106 patients, 37.7% (40) required drain placement during surgery, while 62.3% (66) did not. There was one complication (0.9%), a death due to septic shock secondary to generalized peritonitis. The comorbidities observed in the population included hypertension in 14 patients (13.2%), diabetes in 15 (14.2%), hypothyroidism in 12 (11.3%), and heart failure in five (4.7%).

Conclusions

A potential relationship between patient age and the severity of acute appendicitis was observed. Older patients tend to present with more advanced stages of the disease. Early diagnosis and surgical intervention are particularly beneficial for older patients, reducing the risk of complications.

## Introduction

Aging is an increasing reality, and being a senior adult comes with specific challenges when diagnosing acute appendicitis [1]. Acute appendicitis is one of the most common conditions in surgical practice, and its incidence has been shown to rise with age, peaking after adolescence, with an estimated lifetime risk of 7-8% [2,3]. Recent studies have observed higher rates of mortality and perforation in elderly patients with acute appendicitis, which are often associated with delayed diagnosis, from symptom onset to hospital admission. This delay significantly increases the risk of complications [4]. In senior patients, the clinical presentation tends to be atypical; up to one-fifth of patients seek medical care after three days of symptoms, and between 5% and 10% do so after one week. While abdominal pain is the main symptom, the intensity of the pain in older patients is often less pronounced than in younger individuals, which explains the delay in seeking care [1]. One study found that 30% of patients over 80 years old with abdominal disease who required surgery did not develop an inflammatory response, such as fever or leukocytosis, further contributing to diagnostic delays [5]. Due to the delayed presentation and diagnosis, complications occur more frequently, with appendiceal rupture being one of the most serious, as it is associated with peritonitis, sepsis, and death [6,7].

## Materials and methods

Study design

This is a retrospective, descriptive, observational, single-center study conducted at a tertiary-level hospital. Patient data were collected from the database of the general surgery service for the period spanning from November 2022 to November 2024.

Study population and sample size

The study included all patients who underwent laparoscopic appendectomy during this period and were treated by the same surgical team. Data were gathered from clinical records, and an electronic database was created using Microsoft Excel (Microsoft Corporation, Redmond, WA, USA). A total of 106 patients who underwent laparoscopic appendectomy during the specified period were included in the study, irrespective of gender, age, or comorbidities.

Analyzed variables

The following variables were analyzed: sex, age, Robbins stages, presence of comorbidities, length of hospital stay, use of drainage, and the presence of complications during hospitalization.

## Results

Table 1 presents the demographic data. Exclusion criteria included patients who underwent open appendectomy or were managed conservatively. Demographic variables included sex, age, correlation of the stage with the severity of the disease (as per Robbins classification), comorbidities, hospital length of stay, need for drainage placement, and the occurrence of complications during hospitalization.

**Table 1:** Summary of patient demographics

Parameter	Patients	Percentage
Sex		
Female	49	46.2%
Male	57	53.8%
Age		
Mean	40 years	-
Minimum	13 years	-
Maximum	84 years	-
Comorbidities		
Arterial hypertension	14	13.2%
Diabetes	15	14.2%
Hypothyroidism	12	11.3%
Heart failure	5	4.7%
No comorbidities	60	56.6%
Robbins stage		
Stage 1	5	4.7%
Stage 2	61	57.5%
Stage 3	21	19.8%
Stage 4	19	17.9%
Length of hospital stay		
Mean	2.6 days	-
Minimum	0 days	-
Maximum	9 days	-
Drain placement		
Drain	40	37.7%
No drain	66	62.3%
Complications		
Complications	1	0.9%
No complications	105	99.1%

Of a total of 106 patients who underwent laparoscopic appendectomy during the period between November 2022 and November 2024 in a tertiary care hospital, 46.2% (49 patients) were women and 53.7% (57 patients) were men. In terms of age, the mean age was 40 years, with a minimum age of 13 years and a maximum age of 84 years.

Regarding the progression of acute appendicitis and the different stages of the disease, we can observe that among the patients with stage IV of acute appendicitis, the youngest patient was 53 years old and the oldest patient was 65 years old, with an average age of 59.36 years; on the other hand, among the patients with stage II of acute appendicitis, the youngest patient was 19 years old and the oldest patient was 80 years old, with an average age of 34.83 years. Of a total of 36 patients older than 50 years, 52.7% (19 patients) had stage IV of acute appendicitis, 38.8% (14 patients) had stage III, 8.3% (three patients) had stage II appendicitis, and none had stage I (0%). We can see that most of the patients older than 50 years presented with a more advanced stage of appendicitis, compared to younger patients who tend to present with earlier stages of the disease. The average length of hospital stay was 2.6 days, with one patient discharged the same day of the surgical procedure, and the maximum length of hospital stay was nine days. Of the 106 patients, 37.7% (40 patients) had a drain placed during the surgical procedure, and 62.3% (66 patients) did not need drain placement. From all the patients who had a drain placed during the surgery, their drain was removed prior to discharge, with a minimum hospital length of stay of three days in stages III-IV appendicitis; the rest who did not have a drain placed were stage I-II. Of all patients, 43.4% (46 patients) presented with comorbidities such as hypertension, diabetes, hypothyroidism, and heart failure. The only complication we had was in a 77-year-old patient (0.9%), who presented with intra-abdominal collections due to stage IV appendicitis with generalized peritonitis, who required vasopressors since the time of his surgery and later died because of septic shock.

## Discussion

It was previously believed that the natural course of appendicitis followed a linear progression from inflammation to necrosis and eventually perforation. However, it is now recognized that not all cases follow this path. Gangrenous or perforated appendicitis occurs in approximately 25% of cases, with higher rates observed at the extremes of age, affecting around 40% of patients under 10 years and up to 50% of those over 50 years of age [8]. While the progression of acute appendicitis is traditionally divided into four stages based on intraoperative macroscopic anatomopathological findings, these stages do not always clearly indicate the severity of peritoneal cavity contamination or its systemic repercussions, especially with respect to patient age [9].

Acute appendicitis involves both the appendix and the peritoneal cavity in cases of secondary peritonitis, which can lead to significant local and systemic effects. These must be promptly managed to minimize morbidity and mortality [9,10]. In elderly patients, effective clinical assessment is often hindered by age-related cognitive decline, which compromises communication and the accurate reporting of symptoms. In such cases, support from family members or caregivers becomes essential in providing relevant clinical history [9,11].

Historically, leukocytosis was a key laboratory marker used to support the diagnosis of acute appendicitis and determine the need for surgical intervention. However, the rate of negative appendectomies remained high. Other diagnoses, such as diverticulitis, urinary tract infection, ileitis, colitis, or mesenteric adenopathy, must be excluded before relying on lab parameters, without delaying necessary surgical treatment [12,13]. Although CRP and leukocytosis are widely used as inflammatory markers, their specificity is limited. Other potential markers, such as procalcitonin and interleukins, are not routinely measured in emergency settings [14].

Diagnostic scores like the Alvarado score and the Appendicitis Inflammatory Response score can assist in diagnosing elderly patients, but they are not yet widely implemented in clinical practice [13,15,16]. In our study, all patients underwent blood tests, including CRP levels and complete blood counts. Leukocytosis with elevated CRP was consistently observed; however, no correlation was found between the elevation of these markers and either intraoperative findings or patient age.

Currently, contrast-enhanced abdominal CT is considered the gold standard for diagnosing acute appendicitis in older adults due to its high sensitivity and specificity. In this study, all patients underwent contrast-enhanced CT imaging [16,17]. Although plain abdominal radiographs have limited utility - primarily for detecting free air, signs of obstruction, or foreign bodies - ultrasound remains a valuable alternative, particularly when CT is contraindicated. However, its accuracy depends heavily on operator experience and patient body habitus, which can hinder interpretation [1,2,16].

Elderly patients are more prone to post-appendectomy surgical complications, particularly wound infections and intra-abdominal abscesses, which are also associated with longer hospital stays [4,17]. In our cohort, 36 patients were over 50 years old. Among them, 52.7% (19 patients) had stage IV appendicitis, 38.8% (14 patients) had stage III, and 8.3% (three patients) had stage II. None presented with stage I disease. The length of hospital stay ranged from one to nine days - the longest stay was for a patient who developed intra-abdominal abscesses leading to septic shock and ultimately died on day nine.

Intraoperatively, the appendix was assessed and classified using the Robbins staging system for acute appendicitis [14,17]. The use of surgical drains remains controversial and is typically based on the surgeon’s discretion. In our study, drains were selectively placed in patients with stage III-IV disease to assist in managing undrained or inadequately drained abscesses [15-17].

Our results showed only one complication among the entire study population, including older adults with advanced-stage disease. This low complication rate may be attributed to the consistent use of preoperative antibiotics and prompt surgical intervention via laparoscopy [14,15]. Chronic comorbidities, especially cardiac conditions, play a significant role in increasing morbidity and mortality, likely contributing to the fatal outcome observed in our study [12,14,17].

Limitations

This study has several limitations. It is a retrospective, observational study with a relatively small sample size. Baseline characteristics of older patients, such as functional status and comorbidities, may have influenced intraoperative findings. Additionally, variability in the time from symptom onset to emergency department presentation affected our ability to evaluate postoperative outcomes consistently. Furthermore, atypical symptom presentation and inflammatory marker variability in older adults often delay diagnosis and lead to more severe intraoperative findings and poorer clinical outcomes.

## Conclusions

Our study observed a clear relationship between advanced patient age and more severe presentations of acute appendicitis, as classified by the Robbins staging system. Based on these findings, we conclude that timely diagnosis and surgical intervention are particularly beneficial for senior patients, significantly reducing morbidity and mortality in this population.
